# A large-cohort study of 2971 cases of epulis: focusing on risk factors associated with recurrence

**DOI:** 10.1186/s12903-023-02935-x

**Published:** 2023-04-20

**Authors:** Na Zhao, Yelidana Yesibulati, Pareyida Xiayizhati, Yi-Ning He, Rong-Hui Xia, Xiang-Zhen Yan

**Affiliations:** 1 Shanghai Engineering Research Center of Tooth Restoration and Regeneration, Stomatological Hospital and Dental School of Tongji University, Shanghai, 200072 P. R. China; 2 Biostatistics Office of Clinical Research Unit, Shanghai Ninth People’s Hospital, Shanghai Jiao Tong University School of Medicine, Shanghai, 200011 P. R. China; 3 Department of Oral Pathology, Shanghai Ninth People’s Hospital, School of Medicine, College of Stomatology, National Center for Stomatology, Shanghai Key Laboratory of Stomatology, Shanghai Jiao Tong University, Shanghai Jiao Tong University, National Clinical Research Center for Oral Diseases, Shanghai Research Institute of Stomatology, Shanghai, 200011 P. R. China; 4 Department of Periodontology, Shanghai Engineering Research Center of Tooth Restoration and Regeneration, Stomatological Hospital and Dental School of Tongji University, Yanchang Road 399, Shanghai, 200072 P. R. China

**Keywords:** Epulis, Clinicopathological features, Histological subtypes, Risk factors, Recurrence

## Abstract

**Background:**

To analyze the clinicopathological features of different histological subtypes of epulis, and evaluate the risk factors associated with recurrence.

**Materials and methods:**

A retrospective study including 2971 patients was performed. The patients’ sex, age, location, size, histological subtypes, recurrence information, oral hygiene habits, periodontitis symptoms and smoking history were retrieved from the patient medical records and follow-up information.

**Results:**

Among the 2971 cases, focal fibrous hyperplasia (FFH) was the most common lesion (60.92%), followed by peripheral ossifying fibroma (POF) (29.32%), pyogenic granuloma (PG) (8.08%) and peripheral giant cell granuloma (PGCG) (1.68%). The peak incidence of epulis was in the third and fourth decade of life, with a mean age of 45.55 years. Female predominance was found in all types of lesions with a female to male ratio of 1.71:1. PG had the highest recurrence rate (17.18%), followed by POF (12.98%), FFH (9.55%) and PGCG (8.82%). Histological subtypes were significantly correlated with the recurrence of epulis (*P* = 0.013). Regular supportive periodontal therapy (*P* = 0.050) had a negative correlation with recurrence, whereas symptoms of periodontitis (*P* < 0.001) had a positive correlation with the recurrence of epulis.

**Conclusions:**

Controlling the periodontal inflammation and regular supportive periodontal therapy might help reduce the recurrence of epulis.

## Introduction

Epulis is a clinical term defined as localized gingival overgrowths caused by long-term irritants such as dental plaque, calculus, trapped food, trauma, and iatrogenic factors such as ill-fitting dental appliances. These localized gingival overgrowths are considered as hyperplastic inflammatory reactions, but not neoplasms [1].

Clinically, epulis presents as painless sessile or sometimes pedunculated swellings with smooth or ulcerated surfaces, ranging from a few millimeters to several centimeters. The color of the lesion can vary from bright pink to red [1]. Histologically, the most widely accepted classification nowadays is classified as four subtypes: focal fibrous hyperplasia (FFH), peripheral ossifying fibroma (POF), pyogenic granuloma (PG), and peripheral giant cell granuloma (PGCG) [2, 3]. Only few published studies focused on the relative frequency of the different subtypes of epulis [1, 3–7], and the frequency distribution remains controversial.

Although epulis is benign in nature, it tends to recur. Limited studies reported the recurrence rate of different subtypes of epulis and the risk factors associated with recurrence in patients with epulis remain unclear [5, 7, 8]. Thus, the purpose of this study was to analyze the clinicopathological features of different histological subtypes of epulis, and then to evaluate the risk factors associated with recurrence in patients with epulis.

## Materials and methods

### Ethical Statement

Procedures involving human participants in this study was following the Declaration of Helsinki. The study was approved by the Institutional Review Board of Shanghai Ninth People’s Hospital (SH9H-2022-T106-2, date of approval: 2022.5.12). The informed consent had been waived by Ethics Committee because the retrospective nature of the study.

### Patient cohort and data Collection

A total of 2971 patients who received surgical treatment and were histologically diagnosed with epulis at Department of Oral Pathology, Shanghai Ninth People’s Hospital between January 2010 and March 2022 were included in the study. The clinical information was obtained from the patients’ medical records, including sex, age, location, and size. Patients’ oral hygiene habits, periodontitis symptoms, smoking history and recurrence information were recorded from the follow-up data. The oral hygiene habits included the number of brushing times per day (< 2 times/day, ≥ 2 times/day), the duration of brushing per time (≤ 2 min/time, > 2 min/time), dental floss and interdental brush usage (yes/no), regular supportive periodontal therapy including oral hygiene instruction, scaling, and root planning (yes/no). Symptoms associated with periodontitis included swollen and bleeding gums, mastication weakness and tooth mobility (yes/no). The present study conducted follow-up by telephone interview by Na Zhao, Yelidana YESIBULATI, and Pareyida XIAYIZHATI, who were blinded to histological subtype information and trained at the beginning of the present study. The lesion recurrence (yes/no) was evaluated based on the patients’ reports. 1835 patients had recurrence information.

### Histological evaluation

The removed tissues were fixed in 4% paraformaldehyde and were embedded in paraffin. 4-µm-thick sections were cut from the blocks, and stained with hematoxylin and eosin (H&E). All the cases were reviewed by oral pathologist and categorized into four subtypes: [1] FFH; [2] POF; [3] PG and [4] PGCG based on the histological features.

### Statistical analysis

All data were subjected for statistical analyses using SPSS software 25.0 (SPSS Inc., Chicago, Illinois) and Pearson’s chi-squared or Fisher’s exact tests were used to determine the correlation between the clinicopathological characteristics and the recurrence. All the tests were 2-sided, and p ≤ 0.05 was considered statistically significant.

## Results

### Demographic and clinical features

**Fig. 1:**
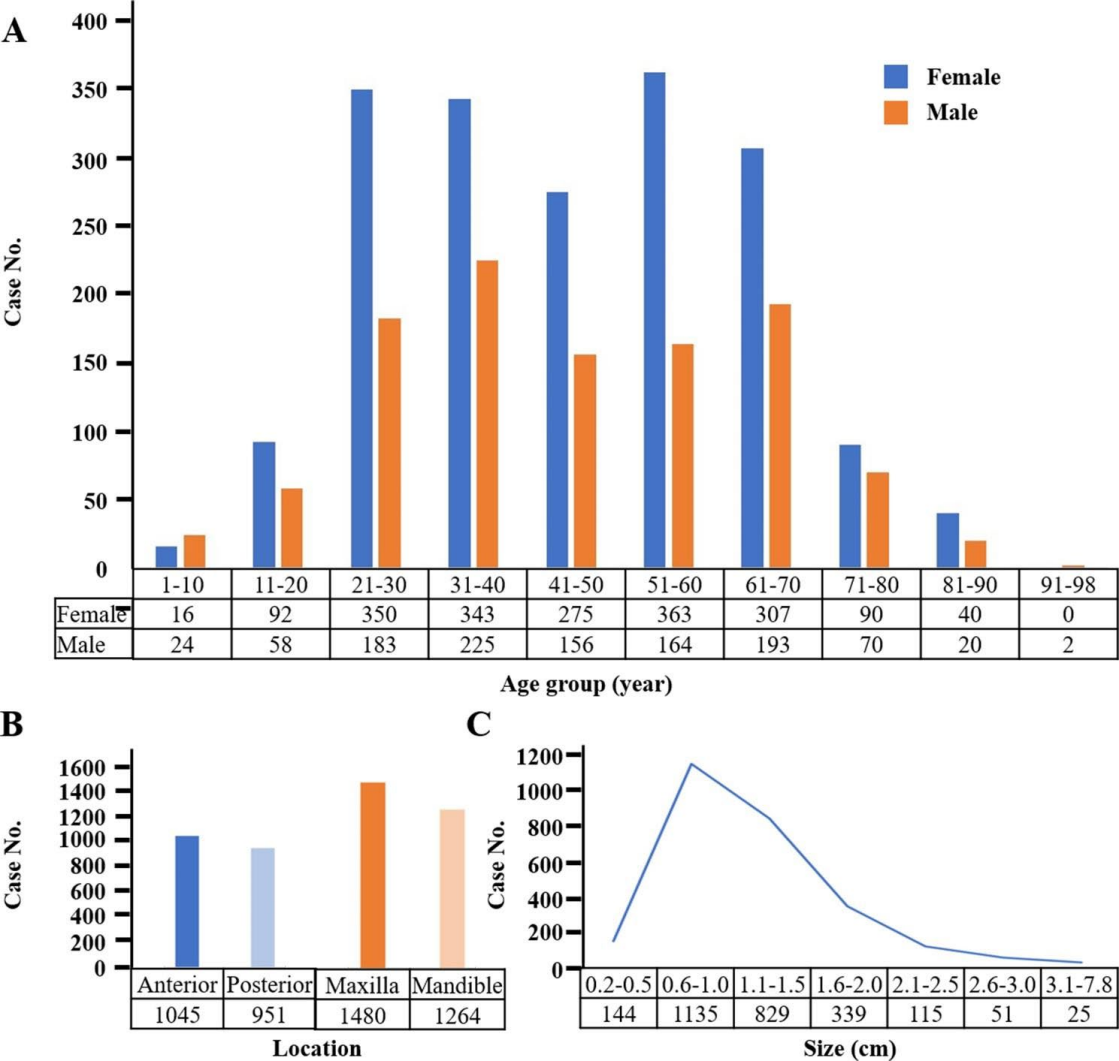
Demographic and clinical features of 2971 cases of epulis. (**A**) Sex and age distribution of 2971 patients. Epulis had predilection for females and the peak incidence was in the third and fourth decade of life. (**B**) Epulis had a slight tendency to occur in anterior region and maxilla. (**C**) The size of most cases was between 0.6 to 1.5 cm

The detailed clinical and demographic data were summarized in Table 1. Among 2971 cases evaluated, 1876 cases (63.14%) were female and 1095 cases (36.86%) were male with a female to male ratio (F/M) of 1.71:1 (χ^2^ = 205.3, *P* < 0.001). The age of patients ranged from 1 to 98 years with a mean age of 45.55 years and a median age of 45 years and the peak incidence was in the third and fourth decade of life (Fig. 1A). For those cases with location information, 52.35% (1045/1996) cases located in the anterior region and 47.65% (951/1996) cases located in the posterior region. The proportion of patients with maxilla and mandible lesions was 53.94% (1480/2744) and 46.06% (1264/2744), respectively (Fig. 1B). Of the 2638 cases for which had size information, the mean size of the lesions was 1.25 cm, ranging from 0.20 to 7.80 cm in diameter, and the diameter of most cases was between 0.6 and 1.5 cm, comprising of 74.45% (1964/2638) of all cases. (Fig. 1C). For those cases with available information, 89.23% had equal or more than twice of brushing per day, 36.70% had more than 2 min for each brushing, 44.99% had dental floss and interdental brush usage, 23.92% had regular supportive periodontal therapy, 18.03% had periodontitis symptoms and 7.28% had smoking history.

**Table 1:** The clinicopathological and demographic features of epulis

Characteristic	No.	%
**Median age, years (range)**	**45 (1–98)**	
**Sex**		
Female	1876	63.14
Male	1095	36.86
**Location**		
Anterior region	1045	52.35
Posterior region	951	47.65
Maxilla	1480	53.94
Mandible	1264	46.06
**Mean size, cm (range)**	**1.25 (0.20–7.80)**	
**Frequency of daily brushing**		
< 2 times	188	6.33
≥ 2 times	1558	52.44
Unknown	1225	41.23
**Duration of brushing per time**		
≤ 2 min	1102	37.09
> 2 min	639	21.51
Unknown	1230	41.40
**Dental floss and interdental brush usage**		
Yes	781	26.29
No	955	32.14
Unknown	1235	41.57
**Regular supportive periodontal therapy**		
Yes	416	14.00
No	1323	44.53
Unknown	1232	41.47
**Smoking history**		
Yes	127	7.28
No	1618	92.72
Unknown	1226	
**Symptoms of periodontitis**		
Yes	318	10.70
No	1446	48.67
Unknown	1207	40.63
**Histological subtypes**		
FFH	1810	60.92
POF	871	29.32
PG	240	8.08
PGCG	50	1.68

### Association between the clinical features and histological subtypes

**Fig. 2:**
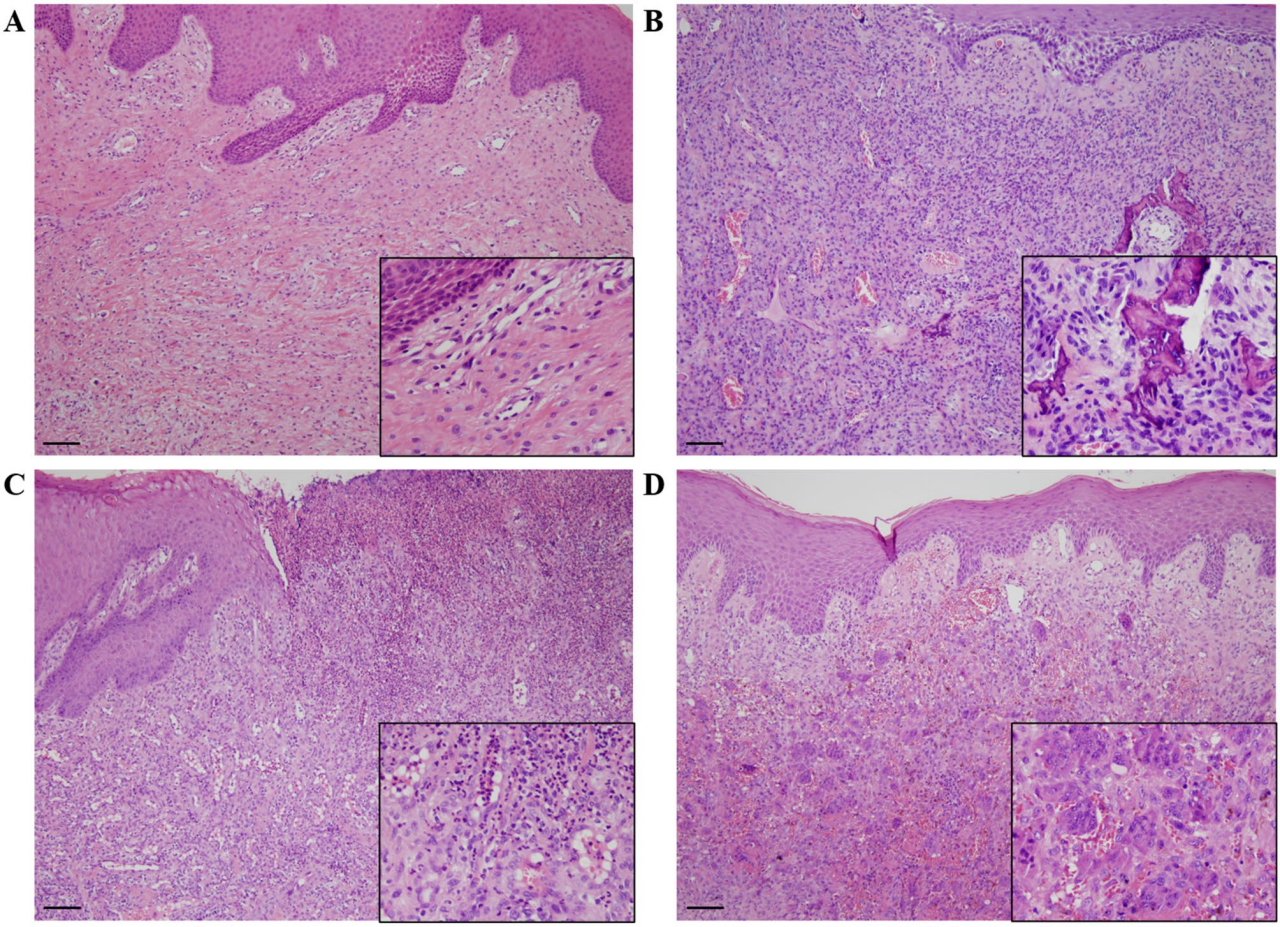
Representative photomicrographs of four histological subtypes of epulis. (**A**) FFH consists of bundles of dense collagen fibers with limited inflammation. (**B**) Cellular fibrous tissue hyperplasia with mineralization presents in POF. (**C**) PG is characterized with the proliferation of endothelial cells and a large amount of inflammatory cell. The lesion is covered by an ulcerated thin layer of stratified squamous epithelium. (**D**) PGCG exhibits focal nodules of multinucleated osteoclast-like cells. Hematoxylin and eosin (H&E) staining, scale bar: 100 μm. Insert: enlarged images

Histologically, epulis were categorized into four subtypes. FFH consists of bundles of dense collagen fibers which are usually arranged a radiating, circular or haphazard shape. Occasionally, the lesion can have chronic inflammatory cell infiltration (Fig. 2A). POF can be recognized by fibrous tissue hyperplasia with variable amounts of mineralization. The mineralized component can be of a variety of types, including woven and lamellar bone, cementum-like material, or dystrophic calcification (Fig. 2B). PG is characterized with the proliferation of endothelial cells, forming small capillary channels or large thin-walled vessels. PG often presents with the infiltration of a large number of neutrophils, lymphocytes, and plasma cells, and is covered by an ulcerated thin layer of stratified squamous epithelium (Fig. 2C). PGCG exhibits focal nodules of multinucleated osteoclast-like cells in vascular and cellular stroma. Similar to PG, PGCG is covered by squamous cell epithelium, which is ulcerated in some cases (Fig. 2D). Other possible features include numerous capillaries growing, hemorrhage, hemosiderin, and mineralized tissue.

FFH was the most common type of lesion (n = 1810, 60.92%), followed by POF (n = 871, 29.32%), PG (n = 240, 8.08%) and PGCG (n = 50, 1.68%). Clinicopathological features based on histological subtypes were summarized in Table 2. All the four subtypes of epulis were more common in females. The female to male ratio of PG was 2.08:1 (χ^2^ = 29.4, *P* < 0.001), which was significantly higher than the overall F/M ratio (1.71:1, χ^2^ = 205.3, *P* < 0.001), FFH (1.69:1, χ^2^ = 117.9, *P* < 0.001), POF (1.69:1, χ^2^ = 57.1, *P* < 0.001), and PGCG F/M ratio (1.63:1, χ^2^ = 2.9, *P* = 0.09).

**Table 2:** Clinicopathological features based on histological subtypes

Characteristic	FFH (No., %)	POF (No., %)	PG (No., %)	PGCG (No., %)	*P* value
**Age, mean ± SD, years**	48.64 ± 18.04	39.21 ± 15.19	44.66 ± 18.19	48.04 ± 17.22	**< 0.001**
**Sex**					
Female	1136 (62.76)	547 (62.80)	162 (67.50)	31 (62.00)	0.543
Male	674 (37.24)	324 (37.20)	78 (32.50)	19 (38.00)	
**Location**					
Anterior region	608 (50.96)	380 (60.13)	45 (33.58)	12 (32.43)	**< 0.001**
Posterior region	585 (49.04)	252 (39.87)	89 (66.42)	25 (67.57)	
Maxilla	900 (54.35)	452 (55.32)	107 (48.42)	21 (43.75)	0.141
Mandible	756 (45.65)	365 (44.68)	114 (51.58)	27 (56.25)	
**Size, mean ± SD, cm**	1.24 ± 0.63	1.18 ± 050	1.53 ± 0.79	1.49 ± 0.61	**< 0.001**

The average age of POF and PG was 39.21 and 44.66 years old, respectively. The average age of PGCG was 48.04 years old, which was older than POF and PG, but close to the age of FFH of 48.64 years. POF tended to affected younger population, which had significant difference from other three subtypes (*P* < 0.001) (Table 2).

PGCG (25/37, 67.57%) and PG (89/134, 66.42%) were more common in the posterior region, while POF was more commonly affected the anterior region (380/632, 60.13%). FFH was distributed with almost equal proportion between the anterior region (608/1193, 50.96%) and posterior region. FFH (900/1656, 54.35%) and POF (452/817, 55.32%) were more common in the maxilla, while the PG (114/221, 51.58%) and PGCG (27/48, 56.25%) were more commonly affected the mandible. The distribution of maxilla and mandible between the four subtypes showed no statistical significance (*P* = 0.141) (Table 2).

The mean size of POF was 1.18 cm (range, 0.20-6.00 cm), which was smaller than that of FFH (1.24 cm, range 0.20-7.80 cm), PGCG (1.49 cm, range 0.60-2.70 cm), and PG (1.53 cm, range 0.40-5.50 cm). The mean size of different types of epulis had statistical significance (*P* < 0.001) (Table 2).

### Risk factors Associated with recurrence in patients with Epulis

Out of 2971 cases, 1835 patients had follow-up information. A total of 207 cases had recurrence, and the recurrence rate was 11.28% (207/1835). PG was the subtype with the highest recurrence rate (28/163, 17.18%), followed by POF (74/570, 12.98%), FFH (102/1068, 9.55%) and PGCG (n = 3/34, 8.82%). Histological subtypes significantly correlated with recurrence (*P* = 0.013). Other clinicopathological characteristics, including regular supportive periodontal therapy (*P* = 0.050) and symptoms of periodontitis (*P* < 0.001) were also significantly correlated with the recurrence of epulis. There was no significant correlation between the recurrence rate of the epulis and age (*P* = 0.413), sex (*P* = 0.461), location (anterior vs. posterior) (*P* = 0.322), location (maxilla vs. mandible) (*P* = 0.596), size (*P* = 0.578), the frequency of daily brushing (*P* = 0.064), the duration of brushing per time (*P* = 0.174), smoking habits (*P* = 0.207), and dental floss and interdental brush usage (*P* = 0.434). The correlation between the risk factors and recurrence was listed in Table 3.

**Table 3:** Risk factors associated with recurrence in patients with epulis

Characteristic	Recurrence (No., %)	No recurrence (No., %)	*P* value
**Age, mean ± SD, years**	42.55 ± 16.01	43.54 ± 16.44	0.413
**Sex**			
Female	72 (10.57)	609 (89.43)	0.461
Male	135 (11.70)	1019 (88.30)	
**Location**			
Anterior region	74 (11.56)	566 (88.44)	0.322
Posterior region	80 (13.42)	516 (86.58)	
Maxilla	103 (11.12)	823 (88.88)	0.596
Mandible	92 (11.95)	678 (88.05)	
**Size, mean ± SD, cm**	1.25 ± 0.61	1.23 ± 0.62	0.578
**Frequency of daily brushing**			
< 2 times	12 (6.38)	176 (93.62)	0.064
≥ 2 times	167 (10.72)	1391 (89.28)	
**Duration of brushing per time**			
≤ 2 min	105 (9.53)	997 (90.47)	0.174
> 2 min	74 (11.58)	565 (88.42)	
**Dental floss and interdental brush usage**			
Yes	85 (10.88)	696 (89.12)	0.434
No	93 (9.74)	862 (90.26)	
**Regular supportive periodontal therapy**			
Yes	32 (7.69)	384 (92.31)	**0.050**
No	146 (11.04)	1177 (88.96)	
**Smoking history**			
Yes	9 (7.09)	118 (92.91)	0.207
No	172 (10.63)	1446 (89.37)	
**Symptoms of periodontitis**			
Yes	52 (16.35)	266 (83.65)	**< 0.001**
No	128 (8.85)	1318 (91.15)	
**Histological subtypes**			
FFH	102 (9.55)	966 (90.45)	**0.013**
POF	74 (12.98)	496 (87.02)	
PG	28 (17.18)	135 (82.82)	
PGCG	3 (8.82)	31 (91.18)	

It was noted that there were 23 patients with multiple recurrence (recurrence more than or equal to 2 times), with 10 cases of FFH (6 females and 4 males), 10 cases of POF (4 females and 6 males) and 3 cases of PG (3 females).

## Discussion

Epulis is a very common oral disease with a prevalence of varying from 5.6 to 20.6% [5, 9–11]. The overgrowth of the gingival has multiple impacts including aesthetic problems, functional disorders, difficulty in chewing and speech, even serious psychological problems [12]. The present report is one of the largest series from single institute thus far. Focusing on the relative frequency distribution of various histological subtypes and analyzing the risk factors associated with recurrence of epulis, the present study provides a reference for in-depth study of the epulis.

In general, epulis had a predilection for female patients, and the results were consistent with Kfir, Buchner and Zhang, who analyzed 741, 1675 and 2439 cases, respectively [1, 3, 4]. Those three literatures reported the F/M ratio as 1.51, 1.49, and 1.40. The reason for this phenomenon was not entirely clear. Hormones, especially female hormones, might be partially contributed. It had been reported that estrogens and other sex hormones exaggerate inflammatory responses in gingival tissue, particularly in pregnancy [13]. The present study showed the average age was 45.55 years and the peak incidence was in the three and four decades of life, which was consistent with Zhang’s report (43.39 years) [4]. However, the average age was much younger in other studies, which showed the average age ranged from 30.0 to 37.7 years old [1, 5, 6]. The discrepancy might be caused by the number of the cases and the regional differences. In the present study, epulis in general were more frequent in the anterior region and maxilla, which was in agreement with those previously reported in most studies [1, 3]. The prevalence in anterior region might be due to the following reasons. Frequent teeth malposition often occurred in the anterior region, leading to the difficulty of oral hygiene maintenance and plaque control. The pooling of saliva provided a rich source of calcium and phosphate, which supersaturated dental plaque and resulted in calculus formation in this area [14, 15]. Though the lesions had a relatively large range of size, most lesions were small, allowing the patient to receive the complete excision in outpatient department.

FFH was the most common histological subtype, comprising 60.92% of all the lesions, followed by POF and PG. PGCG was the least common lesion in the present study, comprising 1.68% of all cases. The similar trend was observed in the previous studies [4, 16–18]. By contrast, this differed from other studies where PG was reported as the most common lesion among all types of epulis [6, 19]. The differences might be attributed to the geographic or ethnic factors, as we found that the relative frequency of four types of epulis followed the same pattern with the previous Chinese investigation (FFH: 61.05%, POF: 17.67%, PG: 19.76%, PGCG: 1.52%) [4]. Other reasons, including unusual terminology and limited case number, might be also contributed [6, 19].

Analyzing sex distribution according to histological subtypes, an interesting finding was noted that all the four types of lesions were more common in female. The high prevalence in female could reflect the role of female hormones and more attention toward dental care. However, the differences presented in different histological subtypes. PG had the strongest predilection for female. Daley et al. concluded that the raised levels of serum progesterone and estrogen had a positive relationship with PG in pregnancies [20]. These hormones made the gingival tissues more susceptible to those chronic local irritants, leading to the development of PG [5, 6, 21]. The expression of estrogen receptors elevated in epulis tissues of pregnancy or non-pregnancy women, suggesting that the development of epulis was estrogen-dependent in a certain degree [22, 23].

POF showed the lowest average age of 39.21 years and the peak incidence was in the third decade. This was in accordance with reports from previous studies which indicated that POF was obviously biased towards younger age group [5, 17]. PG also mainly affected younger patients. By contrast, FFH occurred more frequently in older age groups. In the present study, PGCG and PG were more common in the posterior region, while POF was more commonly affected the anterior region (60.13%), which was consistent with the rate from Buchner et al. (58.3%) [1] and Zhang et al. (64.97%) [4], but higher than that from Kfir et al. (52.6%) [3]. PG had the largest mean size of all four subtypes of epulis, which was in general agreement with Kfir et al. [3].

The reported recurrence rate of epulis has varied widely among different studies. The recurrence rate of 11.28% recorded in the present study was similar to that reported by Babu (10.9%) [7] but lower than that reported in Austria (15.2%) [8]. Interestingly, in a previous study by Effiom et al. from Nigeria, the recurrence rate of epulis was much lower (2.9%) [5]. This discrepancy might be caused by the sample size. The present study had a large sample of 2971 cases and 1835 cases with recurrence information, while other studies consisted of less cases from 92 cases to 314 cases [5, 8]. Only few studies evaluated multiple recurrence of epulis. Of the 207 cases with recurrence in the present study, multiple recurrence occurred in 23 cases (11.11%), which was lower than that reported by Babu (16%) [7]. Multiple recurrence of epulis could be attributed to the failure to remove etiologic factors (e.g., continuous irritations and trauma) and gene regulation [24]. Performing the excision of adjacent periodontal membrane, periostium and alveolar bone, and the root planning could eliminate irritations and avoid the recurrence the epulis [25]. Other factors, including over-expression of anti-apoptotic genes in BCL-2 family and IAP family, could inhibit the apoptosis of gingival tissues, thus leading to epulis [26], and these genetic abnormalities might cause the multiple recurrence of epulis.

To the best of our knowledge, the present study is the largest series analyzing the risk factors associated with the recurrence of the epulis, allowing us to compare the recurrence rate between different histological subtypes for the first time. Different histological subtypes showed statistically significant recurrence rate. PG was the type with the highest recurrence rate of 17.18% and PGCG had the lowest recurrence rate (8.82%). PG frequently developed in pregnant women and the term “pregnancy tumor” had been used in these cases [1], which had high recurrence rate after treatment [27]. Another explanation was that PG were histologically highly vascular proliferative lesions and the vascular endothelial growth factor was related to the angiogenesis and rapid growth of PG [28]. Estrogen could enhance vascular endothelial growth factor production [29], therefore relating to the recurrence. The recurrence rate of PGCG was as low as 1.39% [30]. The low recurrence rate of PGCG might be partly explained by low capacity in neovascularization of granulation tissue. Another reason was that the mast cell count of PGCG was the lowest among all types of epulis [10, 31], while the mast cells were important sources of some proangiogenic and angiogenic factors [32]. Striking difference was reported in the results by Babu and Savage [7, 13], which demonstrated that PGCG had the highest recurrence rate among all types of epulis because the cells of PGCG show high proliferative potential. Further studies are required to reveal the mechanism of recurrence of different subtypes of epulis.

Regular supportive periodontal therapy including oral hygiene instruction, scaling and root planning could significantly reduce recurrence rate compared to the group of patients without it. There was evidence that poor oral hygiene was the main contributing factor leading to recurrence [7, 8]. Verma et al. suggested that the possibility of recurrence could be minimized with proper treatment strategies including standardized supportive periodontal therapy before treatment [33]. Personal oral hygiene status was a key factor in the preservation of periodontal support for a long term [34]. However, in the present study, there was no significant association between the other oral hygiene habits (the number of brushing times per day, the duration of brushing per time, dental floss and interdental brush usage) and recurrence of epulis. This might be explained by the fact that the treatment of dentists removes dental plaque more thoroughly, while the degree of plaque removal of other self-performed oral hygiene habits varies from person to person. Another risk factor for recurrence of epulis was the presence of periodontitis symptoms including swollen and bleeding gums, mastication weakness and tooth mobility. It could be explained that the patients with those symptoms constantly had local irritations.

The limitations of the present study should be noted. First, this was a retrospective study and the data was collected through patient interview, resulted in incomplete records and the information gaps between the researchers and patients. Second, this was a study conducted in a single tertiary hospital, further investigation should be carried out in multicenter to provide thorough conclusions.

## Conclusion

In summary, epulis showed a predilection for female regardless of histological subtypes. POF significantly affected younger patients. PG subtype, patients without regular supportive periodontal therapy, and the patients with periodontitis symptoms had higher recurrence rate. Controlling the periodontal inflammation and regular supportive periodontal therapy might help reduce the recurrence of epulis.

## Data Availability

The datasets used and/or analysed during the current study are available from the corresponding author on reasonable request.

## Abbreviations

FFH: Focal fibrous hyperplasia
POF: Peripheral ossifying fibroma
PG: Pyogenic granuloma
PGCG: Peripheral giant cell granuloma
BCL-2: B-cell lymphoma-2
IAP: Inhibitor of apoptosis protein
